# Oral health system strengthening in fragile and conflict-affected states: A systematic review

**DOI:** 10.7189/jogh-14-04132

**Published:** 2024-06-21

**Authors:** Birke Bogale, Sasha Scambler, Aina Najwa Mohd Khairuddin, Jennifer E Gallagher

**Affiliations:** 1 Faculty of Dentistry, Oral & Craniofacial Sciences, King’s College London, London, UK; 2 Department of Dental and Maxillofacial Surgery, St. Paul’s Hospital Millennium Medical College, Addis Ababa, Ethiopia; 3 Department of Community Oral Health and Clinical Prevention, Faculty of Dentistry, Universiti Malaya, Kuala Lumpur, Malaysia

## Abstract

**Background:**

Oral diseases affect nearly half of the global population, presenting significant challenges in fragile and conflict-affected states. Despite comprising a population of over one billion people, oral health data and comprehensive evidence on oral health system strengthening on these countries are limited. This study, therefore, aims to explore oral health system strengthening in fragile and conflict-affected states by synthesising evidence from relevant global literature.

**Methods:**

We conducted a systematic review of literature across Ovid MEDLINE, EMBASE, Global Health, Scopus, Web of Science, and grey literature databases. The methodological quality of published literature was assessed using the relevant Joanna Briggs Institute critical appraisal tools. The findings were narratively synthesised and presented using the Lancet’s high-quality health system framework.

**Results:**

The review included 23 papers from 12 countries. The evidence documented impacts of armed conflicts, political crisis, pandemics, and natural disasters on oral health systems, and initiatives to strengthen them focusing on the 'foundations' domain. The initiatives included: workforce development and career opportunities; health service platforms such as mobile dental services and teledentistry; integration of oral health into national health systems and emergency responses; contingency planning and adaptability; and effective governance such as financing systems and infrastructures. Collaborative action, both local and international, including monitoring and evaluation were emphasised as key strategies for health system strengthening to ensure an equitable distribution of responsibilities and resources.

**Conclusions:**

Whilst evidence on oral health system strengthening in fragile and conflict-affected states is limited, our findings suggest the need for integrated action, such as mobilising local resources and engaging stakeholders equitably. Further research, with particular focus in the area of processes of care and quality impacts, is necessary to explore comprehensive strategies for strengthening the oral health system.

Oral health, an integral element of a healthy body, is all too often overlooked in both health systems and global health policies [1,2]. Oral diseases are among the commonest noncommunicable diseases despite being largely preventable [2–6]. Hence, an effective oral health system requires prioritising disease control, prevention, and health promotion to preserve the well-being and functionality of the mouth and its surrounding structures [7]. According to Tomar and Cohen [8], attributes of an ideal oral health system include: ‘integration with the rest of the health system; emphasis on health promotion and disease prevention; monitoring of population oral health status and needs; evidence-based; effective; cost-effective; sustainable; equitable; universal; comprehensive; ethical; includes continuous quality assessment and assurance; culturally competent; and empowers communities and individuals to create conditions conducive to health.’ However, human, financial, and material resources remain inadequate in numerous countries, both high- and low-income, resulting in diminished access to oral health care services and unmet treatment needs, particularly in disadvantaged communities [7]. Addressing oral health issues nevertheless becomes significantly more challenging in fragile and conflict-affected states (FCAS), where conflicts and fragility result from a range of disasters, including natural disasters, political instabilities, civil unrest, and pandemics [9,10].

The definition of FCAS lacks consensus. McIntosh and Buckley [11] suggest that it often refers to ‘a fundamental failure of the state to perform functions necessary to meet citizens’ basic needs and expectations, including the assurance of basic security, maintenance of law and justice, and provision of basic services and economic opportunities.’ To measure fragility and potential failure of states, various indices and metrics have been developed based on variable criteria for quantification [12]. Annually, the World Bank group provides an updated harmonised list of 'fragile and conflict-affected situations' to aid the group working in challenging and complex environments, as well as coordinating support in the most vulnerable and marginalised communities. This is based on specific criteria that identify countries and territories with significant levels of institutional and social fragility, as well as those affected by violent conflicts [13]. The 'Fragile States Index' by The Fund for Peace (FFP), which ranks all sovereign United Nations member states based on their vulnerability to conflict or collapse [14], and the Organisation for Economic Co-operation and Development (OECD)’s 'States of Fragility' [15], among others, are also commonly used. Despite variations in terminology across the different indices that have evolved over time, a number of countries consistently appear on the list as fragile or extremely fragile [13–16]. FCAS categorisation and indices have only become prevalent during the past two decades [12]. However, various institutions and databases produce lists of armed conflicts and wars over longer period of time. The Polynational War Memorial provides a comprehensive list of all wars since 1900, compiling data produced by The Peace Research Institute Oslo (PRIO), Correlates of War Project (COW) and Uppsala Conflict Data Program (UCDP) [17]. The wars included in the list with a few exceptions, are based on Small and Singer’s [18] definition of War: ‘being an armed conflict (interstate or intrastate) with at least 1000 battle-related deaths in one calendar year.’

While epidemiological data in the context of FCAS are rare, oral diseases are widely recognised as the most prevalent health conditions globally. Untreated oral diseases alone are reported to affect approximately 3.5 billion people worldwide, which is almost half of the global population [19,20]. The disease burden is higher in populations with lower socioeconomic status, income, or education, and those living in rural areas [20]. Oral conditions are largely associated with significant inequalities among population groups reinforced by inequality and vulnerability driven by poverty, discrimination, and marginalisation [20,21]. Most notably, the determinants, such as food security and nutrition status, often poorer in FCAS, are significant factors in the prevalence and severity of the common oral conditions [2,22]. Conflicts and wars are also recognised as public health emergencies, leading to the evolution of new diseases and exacerbation of existing chronic conditions [23–26]. Besides, FCAS experience significant short- and long-term consequences in their health sectors, including workforce losses due to targeted attacks, detentions, and migrations resulting from conflict and war. Furthermore, low-income countries, encompassing the majority of FCAS [15], have the lowest density of oral health care professionals including dentists (0.55), and dental assistants/therapists (0.07), as compared to the global averages of 3.30 and 2.63 respectively [27]. Often, armed conflicts also deplete resources, leaving health care services without supplies necessitating significant adjustments and restoration [28]. Hence, health care services, including oral health care primarily focus on emergencies, particularly during conflict; and the lack of basic infrastructure, such as medical supplies, water, and electricity, also hinders the provision of care and compromises essential health care services [29]. Besides, the quality of the available care is poor in most circumstances [30]. The situation is also intensified by the lower perception of its importance escalating the burden of oral diseases and their consequences on quality of life over long period of time suggesting a need for a long-term perspective of the impacts and recovery [7].

As indicated by Listl et al. [31], progress in strengthening oral health systems has been slow despite the evident need for improvement. However, there is a growing focus on oral health within global health initiatives and international bodies such as the World Health Organization (WHO) [4]. As a result, various initiatives have been undertaken in formerly and currently FCAS to strengthen their oral health systems, but the evidence is limited and scattered, and there isn’t any comprehensive framework to guide these efforts [31,32]. With over one billion people living in FCAS [15], this is a significant challenge. Therefore, this systematic review aims to explore oral health system strengthening in FCAS by synthesising evidence from relevant global literature. The study employs a contemporary health system strengthening framework, known as the 'high-quality health system framework', conceptualised by the Lancet commission in 2018; and it encompasses three domains, namely, foundations, processes of care, and quality impacts along with their respective components (**Figure 1**) [33].

**Figure 1:**
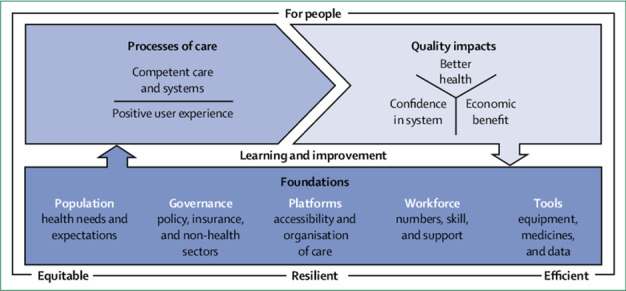
High-quality health system framework [33].

## METHODS

This systematic review has been reported following the Preferred Reporting Items for Systematic Reviews and Meta-Analyses (PRISMA) guidelines [34]. The protocol has been registered on PROSPERO (Reg. number: CRD42022371955).

### Search strategy

We developed the search strategy around two primary concepts: (1) Oral health system; and (2) Fragile and conflict-affected states (Annex 1 in the **Online Supplementary Document**). A compilation of countries identified from a prior systematic review of health system strengthening in FCAS, which were also present at least once in the World Bank's list of fragile and conflict-affected situations since its inception in 2006 were also incorporated in the second concept. This was to increase the likelihood of finding literature not explicitly mentioning terms related to FCAS, based on our repeated trial database searches.

We conducted database searches on Ovid MEDLINE, EMBASE, Global Health, Scopus, and Web of Science databases until the final date of 12 May 2023. The search was extended to include Google, Google Scholar, and relevant international organisations' databases, such as the WHO and World Bank. Grey literature database searches comprised the e-theses online service (EThOS) and Open Access Theses and Dissertations (OATD). We have performed a follow-up database search by the date of 22 November 2023, in addition to forward and backward searching of included papers. We have also contacted authors of papers indicating the potential for further research articles to retrieve a diverse range of literature in this subject area.

### Eligibility criteria

The literature considered in this review comprises both published and grey literature, encompassing reports, reviews, observational and experimental studies, commentaries, book chapters, and other relevant works. Our inclusion criteria focused on literature addressing initiatives for oral health system strengthening and its sub-systems in FCAS. We also considered initiatives that played a role in fortifying the delivery of oral health care services in the long-term. Countries included in the annual list of World Bank’s fragile and conflict-affected situations [13] at least once, and/or those with a history of war since 1900 based on the Polynational War Memorial’s list of wars [17], were deemed eligible. Whilst there were no restrictions on publication years, we limited our selection to English-language literature that was available in full text.

### Screening and management

One reviewer (BB) conducted the initial literature search including forward and backward citation searching, as well as title and abstract screening. All identified records from the database search were exported and deduplicated using Endnote. Further deduplication and screening for title and abstract were performed using the Rayyan software for systematic reviews [35]. Subsequently, BB and ANMK independently conducted the full-text screening of the initially identified articles against the eligibility criteria. BB and ANMK have also conducted methodological quality assessment of the published literature using the selected Joanna Briggs Institute (JBI) critical appraisal tools, including checklists for narrative textual evidence [36], economic evaluations [37], analytical cross-sectional studies [38], studies reporting prevalence data [39], and quasi-experimental studies [40], based on the types of the individual studies. The two reviewers reached a consensus on the application of the eligibility criteria. Additionally, they deliberated on the methodological quality assessment tool and jointly assessed randomly selected papers to ensure consistency in their approach. Any disparities between the two reviewers were resolved through discussion, with input from JEG and SS to achieve consensus.

A MS Excel (Microsoft Inc, Seattle WA, USA) spreadsheet was prepared for the extraction and recording of relevant data, including study ID (author and publication year), title, country, context (e.g. conflict/war, and other humanitarian crises), categories/types of literature, aim/objectives, methods, key findings, and conclusions. BB extracted the data in consultation with ANMK, which were then crosschecked by JEG and SS. Finally, the extracted data were narratively synthesised based on deductive themes, guided by the high-quality health system framework [33]. Each paper was read multiple times, and the data were extracted and organised in line with the key domains and components of the framework accordingly.

## RESULTS

**Figure 2:**
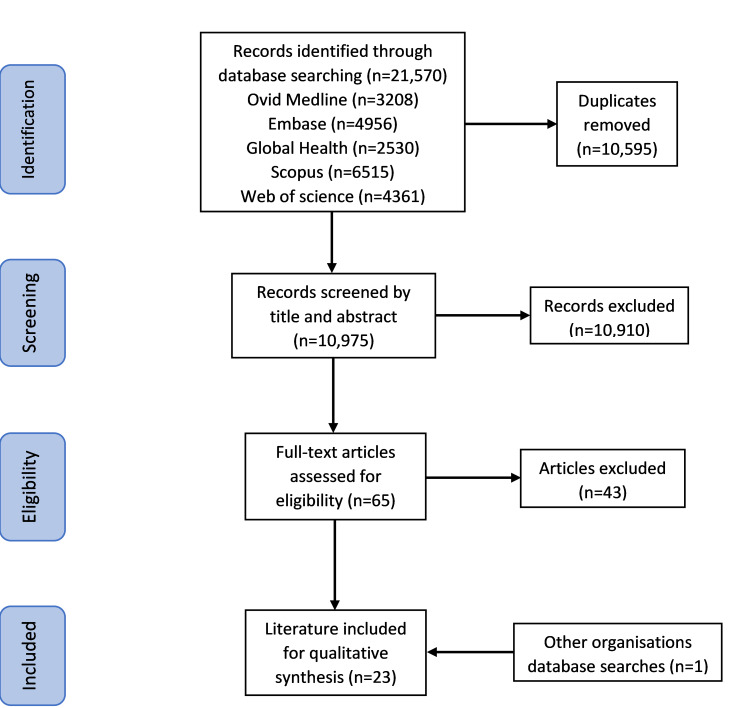
PRISMA flowchart [34].

The database search yielded 21 570 records, with 10 975 screened by title and abstract after removing the duplicates. Of these, 10 910 papers with no evidence of oral health system strengthening initiatives were excluded. Subsequently, 65 full-text articles were assessed for eligibility and 43 papers were excluded. Whilst the latter discussed oral health system strengthening, they did not present any actual evidence of oral health system strengthening initiatives or they reported activities that did not qualify as oral health system strengthening. Thus, 22 papers met the eligibility criteria for inclusion. An additional item of grey literature was identified through searching other organisations' databases. No further papers were identified with the follow-up database search and alternative literature search methods. Therefore, a total of 23 sources, involving published and grey literature produced from 1991–2022 based on the literature search by the final date of 22 November 2023, were included in this study (**Figure 2**).

**Table 1** presents the characteristics of the studies and summarised findings. In terms of the methodological quality appraisal of the 22 included articles using the JBI tools, fifteen papers were ‘narrative textual evidence’ [36] and 12 of which were of good methodological quality. Three papers fulfilled the criteria of economic evaluations [37], of which two were of good quality, and one moderate quality. Two papers employed analytical cross-sectional studies [38] with good and poor methodological quality. The remaining two studies, each reported prevalence data [39], and findings of quasi-experimental studies [40], scoring good and moderate quality respectively. Overall, most papers (n = 16) were regarded as employing good methodological quality, four were considered as moderate, and only two were regarded poor quality based on the scoring criteria of the JBI critical appraisal tools (https://jbi.global/critical-appraisal-tools). All the identified studies were included in the synthesis regardless of their methodological qualities. Among the included studies, nineteen papers specifically examined oral health systems, whilst four incorporated oral health as an additional component of wider health system strengthening initiatives. The papers covered twelve countries included in papers ranging from 1 to 5, namely: Cambodia (n = 5), India (n = 4), South Africa (n = 3), Rwanda (n = 2) and Indonesia (n = 2), as well as Haiti, Mozambique, Turkey, Bosnia and Herzegovina, Croatia, Israel, and Iran (n = 1 each). The papers encompassed findings on the effects of various disaster situations, including conflicts and wars, sociopolitical crises, pandemics, and natural disasters. They also reported efforts to enhance the oral health system, with particular focus on health service platforms (n = 10), workforce (n = 7), financing (n = 4), integration (n = 1), and tools (n = 1).

**Table 1:** Key characteristics of included studies

Author/s, ref No.	Title	Country	JBI critical appraisal tools		Aim/objectives of the papers	Key findings/oral health system strengthening initiatives
			**Study design**	**Methodological quality**		
Estupiñán-Day et al., 2011	Integrating oral health into Haiti’s National Health Plan: from disaster relief to sustainable development	Haiti	Narrative textual evidence	Good	(1) To showcase the oral health response to the crisis in Haiti as a model for how the international community can make a strong, issue-driven effort to fill the gap for services needed. (2) To explain how the Oral Health of Haiti Coalition was made possible through contingency planning according to PAHO’s previously developed Post-Disaster Guidelines for Oral Health. (3) To state that a properly mobilised disaster response can and should pave the way for sustainable development.	The United Nation’s ‘health cluster’ led and coordinated by the PAHO/WHO project involved in the emergency response; and the ‘Oral Health of Haiti Coalition’ was created. Free dental services had been provided by the state dental school as part of the disaster response. The disaster situation created an opportunity to integrate oral health into the primary health care system.
Chher et al., 2019	Improving the Provision of the Basic Package of Oral Care (BPOC) in Cambodia	Cambodia	Quasi-experimental study	Moderate	(1) To provide dental nurses at three health centres with support over a one-year period (including regular supplies of dental materials and monitoring and support visits), and record whether dental care outputs would increase. (2) To compare these outputs with three control health centres which did not receive additional support and supplies (3) To improve cross-infection control practices.	Settings with improved basic package of oral health care had better performance than those without any improvement; and increased monitoring improved compliance with cross-infection control protocols.
Durward and Todd, 1991	Rebuilding the ruins: dental services and manpower in Cambodia	Cambodia	Narrative textual evidence	Good	Not clear	The dental school with the assistant dentist training course was reopened, followed by the doctors of dentistry training programme in 1987. An NGO also assisted in restocking necessary resources such as equipment, materials, books, and journals in the dental training institution.
Mallow et al., 1997	Dental nurse training in Cambodia – a new approach	Cambodia	Narrative textual evidence	Good	Not clear	A dental nurse training was introduced in 1993, and provided to rural nurses who had a one-year basic nursing training. Provincial dentists were engaged in supervision of the dental nurses.
Monguambe and Forgay, 1998	Oral health: Mozambique's response to the challenge	Mozambique	Narrative textual evidence	Good	Not clear	Training programmes for dental therapists and assistants were established in 1975. Previously trained Mozambican dental therapists also took additional courses in Canada and Ireland and assumed leadership and principal tutor’s roles in the provinces and at the new training centre in Mozambique. Regular educational seminars were held in the respective provinces to strengthen the capability of dental workers nationally.
Hackley et al., 2018	A Case Study Optimizing Human Resources in Rwanda’s First Dental School: Three Innovative Management Tools	Rwanda	Narrative textual evidence	Good	To summarise the context and development of the new dental school and describes the three management tools developed by the Human Resources for Health oral health team.	Rwanda’s first school of dentistry was opened in collaboration between three American dental institutions and the University of Rwanda as part of the Human Resources for Health programme. The existing dental therapy programme has also been modified and an independent school in a new university has been established. Three management tools have been developed by the Rwandan and American team which enabled them to understand the successes and challenges in moving toward the planned targets and measure their progress towards collaborative goals.
Seymour et al., 2013	Including oral health training in a health system strengthening program in Rwanda	Rwanda	Narrative textual evidence	Good	Not clear	The 'Human Resources for Health' programme was developed to tackle the country's significant health care challenges by primarily improving existing health education programmes. This initiative involves a partnership with American institutions to ensure that Rwanda's health sciences education system produces a sufficient number of high-quality health care professionals, thus guaranteeing a sufficient supply for the future. The new dental surgery programme was developed applying an innovative ‘diagonal’ curriculum concept.
World Bank, 2012	Indonesia: Health Professional Education Quality Project	Indonesia	Grey literature/report	N/A	Progress report	‘The Health Professional Education Quality Project’ had been implemented in in partnership with the World Bank, governmental and other partner organisations and associations, with the aim of improving the standard of education for health care workers. The ongoing project achieved a number of results such as the involvement of health professional associations in the development of quality assurance framework and establishment of 22 Computer Based Test Centres.
Clarke et al., 2016	Strengthening health professions regulation in Cambodia: a rapid assessment	Cambodia	Narrative textual evidence	Good	(1) Examine the current regulatory system and legal environment for health professions in Cambodia against mandates described in current legislation. (2) Identify, in consultation with stakeholders, areas where the current regulatory system is meeting the requirements of a well-performing regulatory system (and areas where it is not).	The regulatory system could only partially meet Cambodia’s needs. It was not designed with the country’s specific needs in mind. It was also very complex, with considerable duplication and overlap between governance and regulatory arrangements. As a result, a 3-y project applying a health system approach was initiated in partnership with aid organisations, the Ministry of Health and the five independent counsels, including the Dental Council of Cambodia, along with the Medical, Midwives, Nurses, and Pharmacy Councils who were administering the regulatory system with the aim of holistically strengthening the overall health professions’ regulatory system.
Rudolph et al., 1992	A Mobile Dental System in Southern Africa	South Africa	Narrative textual evidence	Good	Describe the evolution, development, and utilisation of a purpose-built mobile dental unit during the period 1988-90.	The mobile dental unit which contains a box trailer with four fully equipped dental operatories and a combined waiting and educational area provided comprehensive care with curative and preventive services by dental auxiliaries based on the primary oral health care approach. The services included examinations, scaling and polishings, group health education, individual oral hygiene instructions, fluoride treatments, fissure sealants, amalgam and composite restorations, extractions, and minor oral surgeries. It also offered students the opportunity for acquiring clinical skills.
Molete et al., 2016	Costs of a school-based dental mobile service in South Africa	South Africa	Economic evaluation	Good	Aim: to undertake a cost-analysis of a school based oral health care programme. Objectives: to estimate the general costs of the programme, costs of oral health care per patient and the implications of providing the services at scale.	The school based oral health care programme in a mobile dental unit service provided oral health screening, fissure sealants, fluoride applications, oral health education, simple extractions, and restorations, also referral when beyond the scope of the mobile units. The personnel costs were the highest cost drivers, followed by vehicles, equipment, and dental materials. The mobile dental unit was cost efficient at 25% allocation of the staff’s time, and a Dental Therapy led service could save costs by 9.1%.
Holtshousen and Smit, 2007	A cost-efficiency analysis of a mobile dental clinic in the public services	South Africa	Economic evaluation	Moderate	To determine the cost-efficiency of the mobile dental clinic utilised in the West Rand region over the first year of implementation.	An accessible and cost-efficient service, with a cost-efficiency ratio of 1.636 (63.6%), yielding a net margin ratio of 0.3889, was provided. The services, including tooth extraction, restoration, oral hygiene procedure and fissure sealants were provided by dentists, dental therapists, and oral hygienists using mobile dental clinic.
Mishra et al., 2014	Dental camp experience in lifeline express (LLE) train among rural population of central, India	India	Narrative textual evidence	Good	To evaluate the application and feasibility of providing screening, diagnosis, preventive dental treatment for rural population through mobile dental unit in lifeline express train from the previous three years in Madhya Pradesh, India.	Dental services were introduced as a trial measure on the Lifeline Express train, and patients received free scaling, fillings, and tooth extraction services. Other surgical procedures including minor surgeries, biopsies and cleft palate repair, and dental x-ray services were also included. The study overall showed that a mobile dental hospital like this can provide an excellent opportunity for oral health care and education in rural populations.
Sandesh et al., 2014	Utilisation of Mobile Dental Vans at Post Graduate Dental Institutions in India	India	Narrative textual evidence	Moderate	To describe the structure, conduct and utilisation of mobile dental vans programmes in consideration to duration of use by the academic institutions for oral health care delivery in rural areas.	Mobile dental vans, mostly operational once or twice a week provided services for 25 to 50, sometimes up-to 100 or more patients in a day providing curative services like amalgam restorations, oral prophylaxis and tooth extractions, and preventive services including topical fluoride applications, along oral health education programmes. The clinic contained an adequate emergency management system together with electricity, water and storage facilities. The service involved chair side assistants and dental hygienists assisting the dentists in most of the programme delivery.
Merali et al., 2014	The Lake Clinic - providing primary care to isolated floating villages on the Tonle Sap Lake, Cambodia	Cambodia	Narrative textual evidence	Good	To demonstrate an innovative mobile clinic model that was developed in 2008 to serve a remote population on the Tonle Sap Lake in Cambodia.	‘The Lake Clinic’ visited remote fishing villages, where no medical services were available, weekly and provided free dental preventive and curative services mainly by volunteer dentists, in addition to primary care, vaccinations, eyecare services, antenatal care and health education. It also transported and accommodated individuals who needed a higher level of care.
Shekhawat et al., 2020	Providing dental services where there are no roads: Lessons from the field	India	Narrative textual evidence	Good	Not clear	The Boat Dental Clinic Programme implemented on an already existing model of ‘Boat Clinics’ for the vulnerable and marginalised communities among the riverine islanders without any access to health care services. It provided dental services installed in two different boats, and successfully provided services including tooth extraction, and filling with amalgam and glass ionomer cement. More complex treatment needs were referred to the nearby community health centre.
Asawa et al., 2015	Utilisation of services and referrals through dental outreach programs in rural areas of India. A two-year study	India	Analytical cross-sectional study	Poor	To evaluate the number of patients, disease pattern and the services provided in the outreach programmes and also effectiveness of patient referral.	Mobile dental clinic used by a dental college and hospital for outreach dental camps to provide dental services to rural communities. Services including patient education, dental examination and consultation, oral prophylaxis, temporary and permanent restorations, extractions, and distribution of free medicines were conducted using two weekly camps and additional days when organised. Referrals to the dental clinics of the institute were provided when the treatment couldn’t be provided at the camp site.
Hariyani et al., 2022	Teledentistry and Online Referral System in Indonesian Primary Health Care Center During the COVID-19 Pandemic: A Narrative textual evidence Review	Indonesia	Narrative textual evidence	Poor	To map the needs and challenges in the application of teledentistry and online referral system encountered by dental health care professionals in Indonesian primary health care centres to provide safe dental health service to the population during the COVID-19 pandemic.	Implementation of teledentistry in the regular dental service during the COVID-19 pandemic has shown promising results, particularly in pharmacological management of dental conditions, as well as detection of dental caries.
Ivankoviæ and Rebac, 1999	Financing of dental health care in the Federation of Bosnia and Herzegovina	Bosnia and Herzegovina	Narrative textual evidence	Moderate	(1) Analysis of the regulations of the Law on Health Insurance and the rights of the insured person to dental health care before, during, and after the 1992-1995 war. (2) Analysis of financial sources for dental health care before, during and after the war. (3) Comparison of dental health care coverage and financing between the Federation of Bosnia and Herzegovina and Western European countries that have, in some of their segments, optimally solved the problem of dental care. (4) Analysis of the future economic development and decentralisation, as well as transformation and privatisation of the dental health care in Bosnia and Herzegovina Federation within the transitory processes in the country.	Initiatives have been taken to reorganise the financial and health systems became fully functional following the peace agreement in 1998. The law on health care and health insurance considered dental health as integral element; it also included prosthetic replacement in the insurance. A law for privatisation of health care was also put in place. The available regular funds for the health care were not sufficient to bring the country to international standards.
Natapov et al., 2016	Does dental health of 6-y-olds reflect the reform of the Israeli dental care system?	Israel	Prevalence study	Good	To collect up-to-date epidemiological data on the dental health of 6-y-olds in Israel to assess if dental reform is already reflected in oral health of young schoolchildren.	Far more treated than untreated caries was generally found after the inclusion of dental care in the health insurance law, despite the relatively unchanged level in total dmft.
Bayat et al., 2006	Dental attendance by insurance status among adults in Tehran, Iran	Iran	Analytical cross-sectional study	Good	To evaluate dental attendance among adults in Tehran, Iran in relation to their dental insurance status.	Dental attendance has been positively related to insurance status in Iran.
Tirgil et al., 2019	Effects of expanding a non-contributory health insurance scheme on out-of-pocket health care spending by the poor in Turkey	Turkey	Economic evaluation	Good	To examine the effect of the 2005 coverage expansion of the Green Card scheme and to address these methodological issues identified in the earlier published studies to demonstrate plausibly the effect of health insurance on utilisation and out of pocket expenditures for the Green Card beneficiaries.	The expansion of a non-contributory health insurance scheme led to significant reductions in out-of-pocket health care expenditures for dental services, as well as diagnostics services, pharmaceuticals, and total medical spending. It has also reduced the incidence of catastrophic expenditures by nearly 50% in people with the largest annual out-of-pocket expenditures.
Pavić Šimetin et al., 2020	Program for Dental Health Advancement in Children „Dental Passport	Croatia	Narrative textual evidence	Good	To present the content and results of the implementation of the ‘Dental Passport’ programme and to analyse them with the focus on the sustainability and coverage of the programme and its implementation in preventive activities and procedures.	In Croatia, a pilot project called the ‘Dental Passport’ has started during the 2017/18 school year with the aim of organising a comprehensive preventive dental examination for all children aged six and 12 as to achieving a lower DMFT, and it later became a national programme. The 2017/18 data showed an increase in the number of examinations, diagnostic and therapeutic procedures.

PAHO – The Pan American Health Organisation Project, WHO – World Health Organization

The findings are organised and presented according to a chosen order of the domains and components of the Lancet's high-quality health system framework.

### Foundations

According to Kruk et al. [33], the ‘foundations’ of high-quality health systems encompass the population with their health needs and expectations; health sector governance and partnerships across different sectors; platforms for delivery of health care; workforce numbers and skills; and tools and resources ranging from medicines to data. This domain yielded most evidence, hence the findings are broken down into the five components below.

#### Workforce

The ‘workforce’ component encompasses all health care workers, planners, and managers, addressing factors such as their quantity and distribution, skills and skill mix, education and training, supportive environment, teamwork, and retention [33]. Seven studies [41–47] shed light on the impact of fragility of states and conflicts on the number and performance of the oral health workforce in four countries (Cambodia, Rwanda, Mozambique, and Haiti), with a focus on initiatives aimed at strengthening them, particularly in the areas of education and training.

In FCAS, especially during conflicts, deliberate or unintentional actions, directly or indirectly influence the number and distribution of the health workforce. Durward and Todd [41] reported that, health services like other institutions, had been completely dismantled in Cambodia during the Khmer Rouge regime between 1975–79, with a purge on health care workers. Only 34 'assistant dentists' and two out of six qualified dentists survived the regime; and neither of the dentists remained in the country. Even before the crisis, Cambodia's health system, particularly oral health, was inadequate to meet the country's needs. The majority of the population relied on traditional dentists trained in an unregulated apprenticeship scheme, providing extensive poor-quality dental treatments like crown and bridge [41]. In post-civil war Rwanda, there were only 92 public oral health care workers, including dentists and dental therapists. All dentists practicing in-country obtained their degrees abroad due to the absence of a training programme for dentists locally. Although dental therapists provided most oral health care services, the existing education programme was inadequate, covering only basic, non-invasive restorative and periodontal procedures, along with simple tooth extractions [42]. Similarly, in Mozambique, there were only 18 dentists and 25 denturists in the country which were all left within one year of independence from Portuguese colonisation in 1975. All the dentists were also trained abroad due to the absence of formal oral health care workers training in the country. Training for auxiliary oral health care workers, conducted under the direction of volunteer dentists from other countries for several years after independence, was halted also in 1984 by the National Directorate of Health until the education system was reorganised [43]. Following the earthquake in Haiti in 2010, many dentists have also left the country [44].

Post-disaster, some governments strongly focus on rebuilding and strengthening their oral health systems along with other public services. According to Monguambe and Forgay [43], Mozambique’s government after independence from Portuguese colony in 1975, prioritised education and health, considering oral health as an integral component. Long-term plans were made to implement a training programme for primary oral health care personnel focusing on prevention and community health. The first oral health care workers' training programmes were then established for dental therapists (‘Agentes’) and assistants (‘Auxiliaries’). Between 1985 and 1988, previously trained Mozambican dental therapists took additional courses in a dental therapy school in Canada and returned to assume leadership roles as oral health care supervisors in all provinces. Thus, Mozambique's National Directorate of Health recommenced the training of oral health personnel, and more dental therapists undertook additional training in Ireland and Canada to assume positions as directors and principal tutors upon their return to the new training centre in the country. However, in 1990, a situational analysis identified a need for support in strengthening the capabilities of oral health care workers, leading to the introduction of regular educational seminars in respective provinces. These seminars, facilitated by oral health teachers from the Institute of Oral Health Sciences and other local health professionals, aimed to enhance working conditions and teaching programmes for future students. To enlarge the workforce in 1992, dental auxiliaries were also upgraded by taking additional courses. Another basic course for new dental therapists was also initiated in 1996 applying problem-based learning [43].

In 2011, the Rwandan Ministry of Health, in partnership with other organisations, initiated a new global health initiative, the 'Human Resources for Health' programme. The initiative aimed to address the country’s greatest challenges of health system and upgrade to high-quality health care by enhancing existing health education programmes. It is a pioneering initiative that encompassed a partnership with American higher institutions and other organisations to ensure that Rwanda’s health sciences education system produces an adequate number and quality of health care professionals to guarantee a sufficient supply in the future [42,45]. The Rwandan minister of health also recognised the shared risk factors and associations between oral health and major noncommunicable diseases, insisting on including dentistry as one of the specialty training areas in the Human Resources for Health programme [45]. As part of the programme, Rwanda’s first school of dentistry opened in collaboration between Harvard School of Dental Medicine, the University of Maryland School of Dentistry, and the University of Rwanda. This programme included a unique project to add a new dental education programme on top of an existing dental therapy programme that involved training mid-level providers with competence in preventive and limited therapeutic care. The three institutions joined together to form the ‘Human Resources for Health oral health team’ and created a pioneering curriculum. Additionally, the existing dental therapy programme has also been modified, and an independent school in a new university has been established [45]. The new dental surgery programme applied an innovative 'diagonal' curriculum concept, blending horizontal interdisciplinary training opportunities with vertical discipline-specific training, aiming to incorporate interprofessional educational opportunities rather than isolated and fragmented professional trainings [42].

To restore dental services, Cambodia initiated training programmes for both dentists and auxiliary dental personnel. The dental school reintroduced the assistant dentist training course, and subsequently, Cambodian dentists reinstated the ‘doctors of dentistry’ training programme in 1987. In 1990, an American non-governmental organisation (NGO) assisted in restocking necessary resources such as equipment, materials, books, and journals in the dental school [41]. In 1992, a new type of primary oral health care worker’s training programme to produce dental nurses was initiated. This training was provided to rural nurses who had one year of basic nursing training and met preset criteria for enrolment. Most Cambodian primary care nurses were male, and they also constituted 89% of the trained primary care dental nurses as a result of the women’s family responsibilities impeding their training. The full-time dental training course, based on a competency-based curriculum, typically provided for four to five months. This course aimed to allow the nurses to acquire the necessary skills before returning to their rural clinics to provide basic dental care, including preventive activities [46]. Trained nurses mostly engaged in dental care on a part-time basis as they continued to carry out general nursing activities for the majority of their time. They were working on a constrained budget, low salary, and excessive commitment with other community health services affecting the provision of dental services [46,47]. Although the programme was expanding with the objective of reaching all provinces, security problems prevented courses from taking place in some provinces. The training was then set to be upgraded to permanent schools with a six-month course. The candidates for the training were selected by the provincial health directors, alongside the Referral Centre (district level) and Health Centre (commune level), where the nurses work [46].

In summary, the findings demonstrated that the situation of conflict and fragility leaves countries with a severe shortage of health care workers, including dentists and dental auxiliary personnel, as evidenced in Cambodia, Rwanda, and Mozambique. These countries in partnership with local and international bodies implemented innovative strategies to address the identified issues. Mozambique prioritised education and health, focusing on training programmes for primary oral health care personnel and further education to upgrade. Rwanda, through the 'Human Resources for Health' programme, established its first school of dentistry, emphasising preventive care, while Cambodia reopened its dental school and introduced new training programmes. These efforts highlight the importance of strategic planning and collaboration in restoring and enhancing oral health workforce post-crisis.

#### Tools

The ‘tools’ component encompasses both software and hardware elements including equipment, supplies, medicines, information systems, culture of quality, use of data, supervision, and feedback [33]. Four studies [41, 45–47] highlighted the challenges and initiatives aimed at strengthening health system tools, with a focus on dental equipment and supervision of oral health care workers in Rwanda, and Cambodia.

The effect of armed conflicts and wars on health facilities has been evident. During the during the Khmer Rouge regime in Cambodia, dental equipment faced various fates, from being collected and stored to being vandalised or discarded into fields and ponds, resulting in both loss and damage [41]. The dental school in the capital suffered extensive loss of books and equipment, posing the need for re-equipping the facility. Despite efforts to reestablish the educational programme, students faced limitations, receiving only lectures without practical experience. Political hindrances to international aid, along with the limited financial support from the government due to commitments to defence and other sectors, exacerbated the shortage of resources and supplies, causing frustration among dental personnel. Besides, the lack of dental facilities in the provinces led the new graduates to shift roles to assistant doctors in the hospitals, while the available facilities in some provinces were also mostly limited to providing emergency care due to lack of supplies and electricity [41]. The effects of the Rwandan civil war on health and educational infrastructures including the oral health care resources have also been detrimental [45].

The absence or limitation of supportive supervision has been identified alongside challenges related to equipment and facilities in Cambodia. Chher et al. [47] reported on dental nurses (medical nurses trained as dental nurses) struggles in Cambodia, citing insufficient equipment and a lack of professional support as barriers to delivering dental services. In response, a pilot experimental study in 2005 examined whether providing dental nurses with sufficient materials, instruments, and support could enhance the delivery of dental services. The study compared settings with an improved basic package of oral health care to those without improvements and demonstrated that adequate resources and higher-level support positively impacted service provision. Increased monitoring further improved compliance with cross-infection control protocols [47].

In 1995 in Cambodia, UNICEF introduced the essential drug programme which included dental local anaesthetic and needles, enabling dental nurses to access supplies through the Ministry of Health central drug supply store [46]. However, nurses often had to purchase some supplies themselves, requiring patients to cover costs and supplement their modest monthly income. Furthermore, provincial dentists played a role in monitoring and supervising the dental nurses post-training, occasionally visiting, or arranging monthly meetings. However, challenges arose due to the nurses' remote locations and the absence of local dentists in some provinces. In certain areas, the lack of interest or involvement by local dentists hindered effective support and monitoring [46].

In summary, the examination of contexts in Cambodia and Rwanda revealed significant challenges hindering the provision of oral health care, such as equipment destruction during armed conflicts and political obstacles to aid. This review identified limited interventions addressing the diverse challenges faced by oral health systems related to dental equipment and supervision in particular. Notably, Cambodia implemented a pilot study to enhance the basic package of care, alongside UNICEF's essential drug programme; and involvement of local dentists in monitoring and supervising dental nurses. The included studies emphasise the continual necessity for sustainable support and strategic planning to address persistent challenges in physical and material resources, as well as supportive supervision.

#### Governance

The health system's 'governance' encompasses various components, including leadership, policies, financing, learning and improvement, and intersectoral elements that involves roads, transport, water and sanitation, electricity, and higher education [33]. Eight studies [42–46, 48–50] shed light on the challenges of health system governance concerning oral health care, and initiatives aimed at addressing them in six countries: Rwanda, Indonesia, Cambodia, Bosnia and Herzegovina, Haiti, and Mozambique.

Several barriers, including administrative challenges, compromised education quality, and limited workforce quality assurance measures, have been identified as hindrances to strengthening the oral health care workforce [45,48,49]. In Rwanda, obstacles such as organisational structure, equipment issues, lack of standardisation, and inadequate infection prevention and control practices impeded the progress of dental training curriculum development [45]. Indonesia faced variations in the quality of health professional education, with many newly established institutions exhibiting poor educational standards [48]. A baseline assessment in Cambodia also revealed shortcomings in key health professional regulation functions, indicating an outdated regulatory system [49].

As a response to these challenges, collaborative projects involving quality improvement and assurance were launched in Indonesia and Cambodia. In Indonesia, the 'Health Professional Education Quality Project' was approved in 2009, and implemented in partnership with the World Bank, governmental organisations and other partner organisations and professional associations. It aimed to improve health care worker education standards through components such as accreditation policy strengthening, national competency-based examination certification for graduates, and a results-based financial assistance package for medical schools [48]. Clarke et al. [49] reported that a three-year project was launched in 2014 in Cambodia to employ a health system approach for the comprehensive enhancement of the overall regulatory system for health professions. The project was collaboratively planned with aid organisations, the Ministry of Health, and the five independent health profession councils responsible for administering the regulatory system. These councils include the Dental Council of Cambodia, as well as the Medical, Midwives, Nurses, and Pharmacy Councils; and the progress, however, hasn’t been reported in the paper [49].

During the process of recovering from disaster and strengthening the health system, the need for coordinated external aid and support are inevitable. Estupiñán-Day et al. [44] noted that in Haiti, integration of oral health in the national health plan, along with coordinated support system, has been an integral element of the process from disaster response to sustainable development in the aftermath of the 2010 earthquake, which was followed by cholera outbreak, and Hurricane Tomas. The Pan American Health Organisation project (PAHO/WHO) led and coordinated the United Nation’s ‘health cluster’ that involved a large number of NGOs. As per the organisation’s recommendation, which resulted from its firm commitment to full preparation and planning for such crises, the ‘Oral Health of Haiti Coalition’ was created. This coalition was established through proactive contingency planning based on PAHO's pre-existing ‘Post-Disaster Guidelines for Oral Health’. The Coalition then engaged representatives of dental associations, dental schools, and foundations striving to meet immediate needs while addressing the long-term objective of rebuilding the oral health system. It was also able to mobilise, quickly and effectively, delivering supplies and services to the areas most impacted by the disaster [44].

In response to disaster situations, various NGOs and high-income countries, including the United States, Canada, and Sweden, have actively supported workforce strengthening efforts in Rwanda, Mozambique, Cambodia, and Indonesia by providing financial support, technical assistance, and project leadership [42,43,45,46,48,49]. Individual volunteers, such as Canadian advisors in Mozambique, have also played crucial roles. However, the sustainability of these projects depends on factors such as the continuity of supplies, strategic planning and country’s ownership of the project. The decision to entrust the educational process to Mozambicans was of fundamental importance in designing the training for the oral health workforce [43]. Similarly, in Cambodia, there was a strategic plan to transition project management to the Ministry of Health, with a concurrent focus on building the capacity of local personnel to take over all aspects of the project, ensuring its long-term viability [43,46]. In Haiti and Mozambique, local governments also worked towards taking over reconstruction, with the possibility of continuous, appropriate, and strategic support from international bodies aligned with government priorities [43,44]. Furthermore, the Human Resource for Health oral health team in Rwanda recognised the need for new tools to document successes, measure impact, and critically track efforts of collaborative projects, ensuring sustainability. Hence, three management tools were developed, focusing on the entire dental school's operations plan, individual faculty members' work plans with deliverables, and a redesigned human resource twinning model. These tools enhanced understanding of successes and challenges, enabling progress measurement towards collaborative goals [45].

Additionally, financial challenges during disasters emphasised the vulnerability of centralised health financial systems and the need for resilient structures. According to Ivankovic and Rebac [50], the 1992–1995 war in Bosnia and Herzegovina disrupted the centralised health financial system, leading to discontinued funding from monetary institutions. Donations were insufficient, particularly for dental services despite their legal inclusion in the health system. Following the peace agreement in 1995, the financial system was reorganised, and the health system regained functionality regardless of its pitfalls. In 1997 the enactment of the law on health care and health insurance, along with the privatisation of companies introduced a distinctive health system, widening the sources of financing and categories of health insurances [50].

In summary, the studies underlined significant challenges in oral health system governance, highlighting barriers such as administrative difficulties, compromised education quality, and limited workforce quality assurance measures. The specific challenges faced in Rwanda, Indonesia, and Cambodia underscore the complexities of developing effective oral health strategies. The findings emphasised the multidimensional nature of health system governance, highlighting the importance of not only establishing and training a workforce and improving physical resources, but also establishing quality assurance. While the involvement of external agencies in supporting health system strengthening initiatives showcases collaborative efforts, it also raises questions about the long-term sustainability, and the need for the country’s ownership of such projects.

#### Platforms

The ‘platforms’ component encompasses assets, including the number and distribution of facilities, public and private mix, service mix, and geographic access; care organisation, including the roles and organisation of community, primary, secondary, and tertiary care, and engagement of private providers; and connective systems, including referral systems, emergency, and community outreach services [33]. Eleven studies [44,51–60] explored some of the challenges in accessing oral health care in FCAS, and the various oral health system platforms created to address those difficulties in six countries: Croatia, Haiti, Indonesia, Cambodia, India, and South Africa.

The study findings indicated that various factors, including geographic locations, resource limitations, sociopolitical situations, pandemics, and natural disasters can significantly impact access to care and constrain health care platforms, leading to poor health outcomes. In Croatia, the 1991 health system reform led to the loss of systematic preventive and curative dental services provided by pedodontics for children, resulting in a high Decayed, Missing, and Filled Teeth (DMFT) and a decline in the quality of paediatric dental care [51]. In Haiti, the earthquake exacerbated existing challenges in dental services, including a low dentist-to-population ratio, destruction of private dental clinics, procurement issues for dental materials, and financial constraints preventing the population from affording oral health care [44]. In addition, the COVID-19 pandemic further complicated patients' access to regular oral health care services in Indonesia [52]. According to Rudolph et al.’s article published in 1992 [53], black rural population in South Africa faced obstacles accessing dental care due to their geographic dispersion over vast and often isolated areas. The urban population also challenged by a critical shortage of oral health personnel in public services, exacerbated by inadequate facilities. Besides, rising costs, reduced government spending, and low oral health priority amidst difficult sociopolitical changes taken place at that time mandated alternative methods in South Africa [53].

Studies included in this review reported the utilisation of mobile dental services, such as boats/ships [54,55], vans/mobile carriages [53,56–59], and a train [60], to deliver dental services in remote and inaccessible areas. Notably, 'The Lake Clinic' in Cambodia, established in 2007, used a ship designed for transporting medical teams and supplies to isolated fishing villages in the Tonle Sap Lake region, providing dental and primary health care, vaccinations, eye care, antenatal care, and health education [54]. In India, 'Boat Clinics' provided dental services to the vulnerable and marginalised communities in the riverine islanders of Brahmaputra River in Assam. This was implemented on existing health care models, offering basic medical care facilities, maternal and child health care, and immunisation using 15 boats. Two dental units were added to these boats in 2015 for a trial run [55]. The Indian 'Lifeline Express' train, the world’s first hospital train, initially focused on general health care and later incorporated dental services in response to an urgent need [60]. Rudolph et al. [53] also described a unique 'mobile dental unit' built for serving underserved populations in both rural and urban South African communities while providing dental students clinical skills and experiences. It has also been reported that mobile dental vans have primarily been employed by higher dental academic institutions, particularly in India, to extend dental services to rural areas [56,57].

The Cambodian Lake clinics, and the Indian boat clinics and hospital train were established by individual non-profit organisations and funded by various local and international entities [54,55,60]. The rest of the mobile services however were collaborative projects funded by individual organisations, dental institutions, local provincial health departments, local businesses, foundations, government bodies, and other institutions [56,59,60]. The findings showed that mobile dental services have commonly provided dental care free of charge [53,54,56,60]. However, in some studies, it remains unclear whether patients pay for services or not [55,57–59]. The services that are predominantly provided by the mobile dental clinics included tooth extractions and restorations using materials like amalgam, composite, or other temporary filling materials. Additional services include distribution of free medicines, scaling and polishing, as well as preventive measures like topical fluoride applications, fissure sealants, oral hygiene instructions, and health education [53–60]. Some mobile services have even provided minor oral surgical services [53], and the hospital train offered cleft palate repairs and dental x-ray services [60]. For complex treatment needs beyond outreach services, referrals to higher institutions have also been made [54,55,57,59,60].

In the provision of mobile oral health services, general and specialist dentists, dental auxiliaries, dental therapists, oral hygienists, and dental undergraduate and postgraduate students have actively participated, either voluntarily or as part of their training [53–55, 58–60]. Additional assistance from local volunteers has been noted in handling registration and logistics [54]. The findings indicated that, dental mobile services, particularly those led by auxiliary personnel, have demonstrated better accessibility and cost-efficiency in schools and local areas [58,59]. The evidence also indicated that mobile dental services can be viable means of providing dental care to rural and underserved populations, as well as mentally and physically disabled patients, effectively utilising teams of oral health auxiliary personnel [53,54,57,60]. However, challenges such as lengthy travel, technical and mechanical issues [53], and difficulties related to the dental workforce, especially dental assistants [55,56], have also been noted.

Beyond mobile dental services, emergency health care responses to humanitarian crisis and the utilisation of e-health to enhance access to oral health care in challenging situations have been reported. During the crisis in Haiti, the state dental school provided free dental services as part of the disaster response, including tooth extractions, temporary restorations, and scaling, until resuming comprehensive care at the same pre-disaster prices [44]. According to Hariyani et al. [52], there was a long-standing discussion on implementation of telehealth in Indonesian primary health care centres to improve health services in remote or isolated areas. Online referral systems and teledentistry have been considered as solutions to provide care in underdeveloped areas and for outreach programmes. During the COVID-19 pandemic, teledentistry emerged as a solution for regular dental services, showing promising results, particularly in the pharmacological management of dental conditions and the detection of dental caries [52].

In summary, this review highlighted the multifaceted challenges impacting access to oral health care in the various contexts of fragility and conflict. The study emphasised the instrumental role of mobile dental services, ranging from boats and vans to unique initiatives like a hospital train, in addressing these challenges, particularly in remote and underserved areas. These mobile clinics, often led by a diverse dental workforce, have demonstrated efficacy in delivering a range of dental services, from basic preventive care to minor surgical procedures. Importantly, their non-profit and collaborative models contribute to providing free or affordable oral health care. Furthermore, the review acknowledged the role of emergency health care responses and e-health solutions, such as telehealth and teledentistry, in overcoming access barriers.

#### Population

The ‘population’ component encompasses health needs, knowledge, health literacy, preferences, and cultural norms of individuals, families, and communities as citizens, producers of better health outcomes, and system users [33]. Five studies [44,47,53–55] shed light on significant population-related barriers to oral health care and the innovative initiatives devised to overcome these challenges in four countries: Cambodia, South Africa, India, and Haiti.

Limitations in community awareness and cultural barriers posed significant challenges in providing care and improving health outcomes in FCAS. In addressing oral health challenges, Cambodia faced obstacles related to limited community awareness about oral diseases and prevention [47]. Similarly, in South Africa, cultural barriers, influenced by knowledge, beliefs, and attitudes toward oral health care, posed challenges to utilisation of care in both rural and urban areas. This was particularly evident when introducing mobile dental services to cater to the urgent needs of underserved populations, with efforts involving students in practical community-oriented learning [53]. In Cambodia, the lake clinic involved community volunteers for patient registration and logistics. It also established a village health volunteer system to monitor the local community’s health [54]. The boat clinics in India prioritised community participation by informing the community about the services and distributing posters and pamphlets in the local language; engaging local health workers also facilitated the provision of the oral health care services [55]. Furthermore, in Haiti, stakeholders emphasised a self-sustaining model rooted in the local community, aiming to empower local talent through training and knowledge transfer in oral health practices [44].

In summary, the findings underscored challenges related to limited awareness of the population and cultural barriers in oral health care provision and outcomes in FCAS. They also emphasised the critical roles of customised local community involvement and innovative approaches in oral health initiatives, including awareness campaigns and culturally sensitive strategies. Engaging local communities not only improves access but also contributes to the sustainability and effectiveness of oral health initiatives. The incorporation of students and local volunteers further strengthens these efforts, highlighting the interrelationship of community engagement and successful oral health care provision.

### Processes of care

The 'processes of care' domain includes two components: 'competent care and systems,' which encompasses elements such as capable systems, evidence-based, and effective care; and 'positive user experience,' which includes elements of respect and user focus [33]. Six studies [44,46,51,55,57,60] presented evidence on initiatives related to aspects of 'competent care and system,' focusing on integration, disease prevention, and referral in four countries: Haiti, India, Croatia, and Cambodia.

Integration of oral health services into an established system and adapting available tools have been crucial strategies for disaster response, further enhancing access to care. According to Estupiñán-Day et al. [44], following the crises in Haiti, the ‘PAHO’s Post-Disaster Guidelines for Oral Health’, applicable in any emergency setting, underwent targeted adaptation to address the country's unique needs. This tailored approach enabled the oral health coalition to coordinate a cohesive and sustainable disaster response. The authors underscored the vital role of predefined guidelines, highlighting that the absence of such guidance could have resulted in oversights in deploying essential oral health services and equipment within the broader health cluster response. With the support from the PAHO, the coalition initiated the steps toward integrating oral health into the primary health care system, expanding the basic package of health services [44]. Integration of services has also been implemented to mobile dental clinics in India, where the incorporation of oral health practices aims to promote oral health and reduce disparities in accessing care within underserved populations [55,60].

Community health services, connected to higher-level health care through referral systems, have been recognised for enhancing access to care. The 'Dental Passport' project in Croatia emerged as a significant initiative targeting the reduction of DMFT in children. Originating as a comprehensive preventive dental examination in the 2016/2017 school year, it evolved into a national programme in 2017/2018, showcasing the sustainable implementation of dental examinations and preventive procedures for schoolchildren. The pilot project involved the referral of all 6- and 12-year-old children by a school doctor to receive regular check-ups, health education, fluoride tooth brushing, and the documentation of essential epidemiological data [51]. As noted by Asawa et al. [57], provision of referral at outreach services oral health care awareness and motivation programmes in India significantly influenced service utilisation, with a higher proportion of females benefiting from the programmes upon referral [57]. However, challenges persisted in Cambodia, as highlighted by Mallow et al. [46], who underscored the difficulties dental nurses faced in obtaining assistance in treating patients due to the absence of a functional referral system from health centres to hospitals.

In summary, the evidence underscored the importance of predefined and adaptable guidelines in post-disaster oral health responses, along with long-term strategic health system strengthening initiatives as evidenced in Haiti. The integration of oral health into broader health care models and innovative projects like the Croatian school dental programmes showcased promising advancements in promoting oral health and addressing existing disparities. The critical role of effective referral systems, highlighted by both positive and challenging experiences in various contexts, emphasised the necessity for comprehensive strategies to ensure the success and sustainability of oral health programmes. Overall, these experiences underscore the importance of adaptive strategies and collaborative efforts in strengthening the process of oral health care and disaster response systems.

### Quality impacts

The 'quality impacts' domain comprises three components: 'better health,' which assesses the level and distribution of patient-reported outcomes; 'confidence in system,' which encompasses satisfaction, recommendation, trust, and care uptake and retention; and 'economic benefit,' encompassing the ability to work or attend school, economic growth, reduction in health system waste, and financial risk protection [33]. Three studies [61–63] provided insights into aspects of the 'better health' and 'economic benefit' components, focusing on oral health insurance and its implications for improved access to dental care, oral health outcomes, and financial risk protection in three countries: Israel, Iran, and Turkey.

Dental insurance has demonstrated a substantial impact on dental service attendance and outcomes in Israel and Iran. Natapov et al. [61] noted a pivotal development in Israel's 'National Health Insurance Law' of 1994, which initially excluded dental services but was amended in 2010 to incorporate dental care for children up to eight years old. This age eligibility gradually increased, reaching 12 years by 2013. The local governmental body, recognising its responsibility for community-based primary oral health care for schoolchildren, increased the budget for school dental services, potentially contributing to improved oral health outcomes. Whilst the overall decayed, missed and filled teeth (dmft) level remained relatively steady, a significant shift was observed – more treated caries compared to untreated caries—following the inclusion of dental care in the health insurance law [61]. In Iran, a country with a low dentist-population ratio, a cross-sectional study using phone interviews to assess dental attendance by insurance status among adults found a positive relationship between dental attendance and insurance status. Iran had two dental insurance systems – public and commercial – with companies under the Labour Law mandated to insure their employees [62].

Tirgil et al. [63] investigated the impact of expanding a non-contributory health insurance scheme on out-of-pocket health care spending by the poor in Turkey. As part of a broader health system reform for universal health coverage initiated in 2003, the country expanded the Green Card scheme, which is a government-funded health insurance initiative for the poor, between the years 2003 and 2006. This expansion included coverage for dental services, significant reductions were observed in out-of-pocket expenditures for dental care, as well as for diagnostics services, pharmaceuticals, and total medical spending. The scheme also resulted in a nearly 50% reduction in the incidence of catastrophic health expenditures for individuals with the highest annual out-of-pocket expenses [63].

In summary, the evidence demonstrated the influence of dental insurance and financing on health care attendance and outcomes in diverse contexts. Israel's transformative amendment to the National Health Insurance Law notably impacted oral health outcomes by increasing the proportion of treated oral diseases. In Iran, the positive correlation between dental attendance and insurance status emphasised the role of insurance in promoting oral health care utilisation. Meanwhile, Turkey's expansion of the insurance scheme, including dental services, as part of its universal health coverage reform, resulted in significant reductions in out-of-pocket expenditures demonstrating the potential of comprehensive health insurance initiatives to alleviate financial burdens on individuals. These insights collectively underscore the pivotal role of oral health policy and its inclusion in national policies is crucial for enhancing access to care and the quality impacts, ultimately leading improved health outcomes.

## DISCUSSION

This systematic review illustrates the complex landscape of oral health and care within FCAS, providing insights into the challenges, facilitators, and initiatives aimed at strengthening the oral health system. Despite the Lancet's high-quality health system framework being designed for the broader health system, it effectively facilitated data synthesis and presentation in this review. Notably, evidence on oral health system strengthening is limited. Nevertheless, almost three-quarters of the included papers in this study demonstrated good methodological quality, and most were authored by individuals with a hands-on experience of the reported oral health system strengthening initiatives. These observations suggest that confidence can be placed in the findings of this review. Based on the findings, the available evidence predominantly focused on the 'foundations' domain, similar to other health system areas [33]. The sparse evidence in the 'processes of care' and 'quality impacts' domains, especially the latter, primarily originated from countries that have maintained relative stability over a period of time including Croatia, India, Israel, Iran, and Turkey which had made significant progress in building or rebuilding their health systems. This contrasts with situations in currently or recently FCAS where a system has been decimated, and the prospect of achieving quality seems to be a long journey that began with rebuilding the foundations. This may suggest a predisposition of the Lancet’s high-quality health system framework [33] towards states of stability and its limited application in FCAS, particularly during and soon after disaster situations. Nonetheless, the framework can be a valuable tool for future health system planning, recognising that its application may require time for recovery and stability in countries and the health systems. Out of the twelve countries included in the review, only four (Cambodia, Haiti, Mozambique, and Bosnia and Herzegovina) have been listed in the World Bank's fragile and conflict-affected situations, with Haiti consistently included in the list since its inception in 2006 [13]. The eligibility of the remaining countries was determined by The Polynational War Memorial's list of 'Wars since 1900' [17]. Countries like South Africa and Rwanda, marked by histories of significant armed conflicts and political crises, could align with contemporary fragile and conflict-affected situations criteria; they would have been classified as such if the measurement and indices were established during or before the 20th century.

The body of evidence suggests that the oral health system in FCAS is marked by a lack of facilities and a critical shortage of resources, including workforce, finance, and supplies in public services [41,45,47,53]. Specific instances in countries like Croatia, Haiti, and Indonesia underscore the vulnerability of oral health and care to systemic disruptions [44,51,52,64]. Conflicts typically deplete a significant portion of a country's resources, placing the health system in competition with other government priorities, notably pronounced in oral health care services, even within the broader health system [41,53]. Furthermore, national health systems often become intentional or unintentional targets during conflicts, compelling health care workers to migrate in disaster situations [65–69]. This phenomenon has been observed in countries like Cambodia, Mozambique, Rwanda, and Haiti, where the already sparse health care workforce has faced casualties and migrations [41–44]. As evident in the reviewed studies, the predominant focus has been on the production and enhanced performance of oral health care workers. This emphasis may arise from the oral health sector's attention being primarily directed at the initial stages of crises, where prioritising the replacement of lost workforce and resources is imperative. However, workforce retention takes precedence in numerous other health system strengthening studies. Studies related to oral health workforce in high-income countries such as the UK also focused on workforce retention and sustainability [70,71]. This contrasts with countries categorised as FCAS and/or low-and-middle-income countries, where the production of the oral health workforce is a priority due to its pressing scarcity while retention should also be sought after [72,73].

Fragility, often observed nationally, can manifest more severely, or be localised in specific geographic areas even with non-fragile states [11,74]. This is exemplified by challenging geographic locations such as riverine islands and isolated fishing villages, as well as sociopolitical situations that isolate communities and deprive them of basic services [53–55]. This highlights the diverse dimensions of access, emphasising that strengthening physical infrastructure and other health system resources, including the workforce, finance, and tools, is insufficient without effective means to reach the population, whether physically or virtually. The review also stresses the necessity for adaptability in oral health care services, showcasing the potential of various platforms, including mobile services, where health care is brought directly to the public, and the use of innovative technologies to enhance access even in challenging circumstances. Integrated care is recognised as a vital strategy for enhancing access and optimising resource utilisation, with the integration of oral health into primary health care services being an ongoing subject of discussion [75–77]. Whilst the evidence supporting integration is limited, this review showcases instances where oral health initiatives were presented either as independent programmes or integrated into broader health care services, either from their inception or as an addition to existing initiatives. Some mobile health services and outreach programmes have also been incorporated into training institutions and linked to high-level health care providers through referrals. Dental schools have engaged in such projects, contributing to society while providing real-life clinical experiences to their students, which underscore the importance of comprehensive utilisation of scarce resources FCAS [53,56,57]. However, the quality and effectiveness of care in mobile dental services remain largely unknown, despite playing a vital role in improving access to dental care, particularly by reaching vulnerable communities showcasing the need for considering the potential impacts while planning initiatives.

Certain oral health strengthening programmes included in this review have adapted existing initiatives to suit local contexts, whilst others have innovatively evolved as resource-efficient solutions. In terms of programmes to strengthen workforce, strategies such as the diagonal approach in Rwanda's dental education curriculum, problem-based learning in primary oral health care worker training in Mozambique, and the Cambodian rural nurses training to provide basic oral health care services, exemplify tailored responses to urgent needs [42,43,45,46]. Additionally, the importance of ready-made guidelines that are adaptable to the context and situation at hand has been demonstrated in Haiti, facilitating disaster response and long-term health system strengthening initiatives [44]. The PAHO, the regional office for the WHO in the Americas, has produced the Post-Disaster Guidelines for Oral Health in 2010 aiming to serve as a multi-sectoral framework to organise and coordinate a disaster relief effort of humanitarian actors [78]. This guideline, besides serving as a crucial tool to facilitate coordination of emergency response and health care provision, laid a foundation for further health system strengthening and resilience. It also demonstrates the importance of planning and preparation for any inevitable disaster situations [44,78]. This review also highlights opportunities presented by disaster situations for implementing previously conceived or new initiatives, as demonstrated by the application of teledentistry in Indonesia during the COVID-19 pandemic, and integration of oral health in the primary health care system during Haiti's health system reconstruction after natural disasters, emphasising the importance of strategic initiatives [44,52].

Most of the identified initiatives aimed at strengthening oral health systems have originated within the respective countries. Several developed countries' governments, NGOs, universities, volunteer individuals, and international development organisations have actively participated in the process, particularly in workforce strengthening initiatives in Rwanda, Mozambique, Cambodia, and Indonesia [42,43,45,46,48,49]. Some of the mobile health services initiatives were also initiated by volunteers and NGOs independently of the broader health system to reach vulnerable and isolated communities [53,56,57]. This may raise questions about the continuity of the programmes and coordination with other potential initiatives in the area. However, it demonstrates the substantial role external actors play in FCAS, particularly in transitioning from disaster situations, by addressing emergencies and facilitating the long-term restoration of health systems. Nevertheless, the limited evidence on local community engagement in oral health system strengthening programmes or its oversight in the literature indicates a potential gap, not only their engagement, but also the possibility of some initiatives not being reported in general. Additionally, the challenges faced by external actors in engaging with certain countries, often attributed to political situations, particularly as observed in Cambodia, highlight barriers to effective collaboration [41]. The projects included in this review, however, were mostly designed to transition the responsibilities to the government and local agencies [43,44,46]. Still, the sustainability of these programmes remains uncertain, potentially resulting in a decreased focus on oral health in later stages and the risk of exclusion from health system reforms, as observed in Croatia [51]. Moreover, the optimal role for external agencies and individuals seeking to contribute lies in assisting with the development and implementation of strategies aligned with the goals identified by the country itself. This involves fostering ownership, mutual support, and ongoing monitoring to ensure meaningful and lasting impact [45].

This study acknowledges several limitations. 1) Country selection bias: our inclusion criteria were not designed to favour or discriminate against countries based on their temporal status of fragile and conflict-affected contexts, potentially leading to a missed consideration of studies conducted in the later stages of recovery. Nevertheless, the list of countries drawn from our previous study, incorporated into the search strategy, enhanced the possibility of retrieving essential papers that might not have surfaced in the search but could offer valuable insights. 2) Contextual variations: the included papers explored different contexts with temporal variations, presenting challenges in synthesis. Nevertheless, the study shows that the context of conflict and fragility, along with its consequences on people and health systems, has remained relatively consistent over time, regardless of its cause. 3) Diverse source types: the inclusion of various sources, such as research articles with distinct methodologies, personal experiences, and reports, posed challenges in methodological quality assessments. The scarcity of written resources on oral health system strengthening and the varying contextual situations contributed to this diversity. However, some papers, authored by individuals with firsthand experience, enhanced the reliability of the data despite potential subjectivity. 4) Language restriction: due to resource limitations, the study imposed a language restriction, potentially excluding relevant papers written in languages other than English. Nonetheless, the evidence was synthesised comprehensively, considering all available options to construct a compelling narrative.

## CONCLUSIONS

This systematic review presents the growing, but limited, body of evidence on oral health system strengthening in FCAS drawing on the Lancet’s high-quality health system framework. It synthesises insights from various initiatives aimed at strengthening oral health systems offering potential applications across different settings. This review encompassed currently FCAS, and those with a history of fragility and conflict, which have made progress in strengthening or rebuilding their health systems during a period of relative stability.

The challenges encountered by nations endeavouring to enhance the oral health of their populations are both extensive and variable. Consequently, the findings support the necessity for context-specific responses utilising local resources and the principles of ongoing community engagement to develop resilient and responsive oral health systems, on a journey towards improved oral health outcomes. Effective policy, contingency planning, adaptability, integration, and partnerships played pivotal roles in effective and sustainable oral health system strengthening initiatives.

The concentration of evidence on the foundations of health systems suggests that continuous effort is required beyond providing physical, financial, and technical resources at all levels to build a high-quality oral health system. Furthermore, the limited evidence, particularly the processes and quality impacts, also implies the need for targeted research to inform and contribute to formulating effective policy and strategy in strengthening the oral health systems over time.

## Additional material


Online Supplementary Document
