# Supplementary material for: Oral health system strengthening in fragile and conflict-affected states: A systematic review
Source: J Glob Health. 2024 Jun 21;14:04132. doi: 10.7189/jogh-14-04132 (PMC11190633; doi:10.7189/jogh-14-04132)
Supplement: Online Supplementary Document [file jogh-14-04132-s001.pdf]

Annex 1

Supporting information: Search strategy

I. Scopus

(TITLE-ABS-KEY ((oral W/3 (health OR healthcare OR care)) OR (dental W/3 (health OR healthcare OR care OR system? OR service?)))) AND (TITLE-ABS-KEY (("conflict affected") OR ("post conflict") OR ((fragil* OR conflict* OR weak OR war? OR ("war torn")) W/3 (state? OR countr* OR territor* OR region* OR setting? OR context? OR situation?)) OR (humanitarian*) OR (complex W/2 (emergency OR emergencies)) OR (afghanistan OR angola OR bosnia OR herzegovina OR ("Burkina Faso") OR burundi OR cambodia OR ("Central Africa?") OR congo OR ("Cote d'Ivoire") OR ("Ivory Coast") OR djibouti OR eritrea OR ethiopia OR gambia OR guinea OR haiti OR iraq OR lao? OR lebanon OR liberia OR libya OR mali OR mozambique OR myanmar OR burma OR nepal OR niger OR nigeria OR ("Sierra Leone") OR ("Solomon Island?") OR somali* OR sudan OR syria OR tajikistan OR timor OR togo OR yemen OR zimbabwe OR cameroon OR colombia OR croatia OR guatemala OR india OR indonesia OR israel OR pakistan OR palestin* OR philippines OR russia OR rwanda OR ("South Africa") OR turkey OR uganda OR chechnya OR georgia OR chad OR comoros OR iran OR kosovo OR ("Sri Lanka") OR ukraine))) AND (LIMIT-TO (LANGUAGE , "English"))

II. Web of Science

1. TS=((conflict-affected OR post-conflict OR postconflict OR ((fragil* or conflict* or war? or war-torn) NEAR/3 (state? or countr* or territor* or region? or setting? or context? or situation?)) OR complex-emergency OR complex-emergencies OR humanitarian*))
2. TS=((Afghanistan or Angola or Bosnia or Herzegovina or Burkina Faso or Burundi or Cambodia or Central Africa? or Congo or Cote d'Ivoire or Ivory Coast or Djibouti or Eritrea or Ethiopia or Gambia or Guinea or Haiti or Iraq or Lao? or Lebanon or Liberia or Libya or Mali or Mozambique or Myanmar or Burma or Nepal or Niger or Nigeria or Sierra Leone or Solomon Islands or Somali* or Sudan or Syria or Tajikistan or Timor or Togo or Yemen or Zimbabwe or Cameroon or Colombia or Croatia or Guatemala or India or Indonesia or Israel or Pakistan or Palestin* or Philippines or Russia or Rwanda or South Africa or Turkey or Uganda or Chechnya or Georgia or Chad or Comoros or Iran or Kosovo or Sri Lanka or Ukraine))
3. #1 OR #2
4. TS=((oral NEAR/3 (health or healthcare or care))) OR TS=(dental NEAR/3 (health or healthcare or care or system? or service?))
5. #3 AND #4 and English (Languages)

III. OVID Medline

1. exp Oral Health/
2. exp Dental Care/
3. (oral adj3 (health or healthcare or care)).ti,ab,kw.
4. (dental adj3 (health or healthcare or care or system? or service?)).ti,ab,kw.
5. or/1-4
6. (conflict adj2 affected).ti,ab,kw.
7. post conflict.ti,ab,kw.

8. postconflict.ti,ab,kw.
9. ((fragil* or conflict* or weak or war? or war NEXT torn) adj3 (state? or countr* or territor* or region* or setting? or context? or situation?)).ti,ab,kw.
10. humanitarian*.ti,ab,kw.
11. (complex adj2 (emergency or emergencies)).ti,ab,kw.
12. Afghanistan.ti,ab,kw.
13. (Bosnia or Herzegovina).ti,ab,kw.
14. Burkina Faso.ti,ab,kw.
15. Cambodia.ti,ab,kw.
16. Central Africa?.ti,ab,kw.
17. Angola.ti,ab,kw.
18. Burundi.ti,ab,kw.
19. Cambodia.ti,ab,kw.
20. Central Africa?.ti,ab,kw.
21. Congo.ti,ab,kw.
22. Cote d'Ivoire.ti,ab,kw.
23. Ivory Coast.ti,ab,kw.
24. Djibouti.ti,ab,kw.
25. Eritrea.ti,ab,kw.
26. Ethiopia.ti,ab,kw.
27. Gambia.ti,ab,kw.
28. Guinea.ti,ab,kw.
29. Haiti.ti,ab,kw.
30. Iraq.ti,ab,kw.
31. Lao?.ti,ab,kw.
32. Lebanon.ti,ab,kw.
33. Liberia.ti,ab,kw.
34. Libya.ti,ab,kw.
35. Mali.ti,ab,kw.
36. Mozambique.ti,ab,kw.
37. Myanmar.ti,ab,kw.
38. Burma.ti,ab,kw.
39. Nepal.ti,ab,kw.
40. Niger.ti,ab,kw.
41. Nigeria.ti,ab,kw.
42. Sierra Leone.ti,ab,kw.
43. Solomon Island?.ti,ab,kw.
44. Somali*.ti,ab,kw.
45. Sudan.ti,ab,kw.
46. Syria.ti,ab,kw.
47. Tajikistan.ti,ab,kw.
48. Timor.ti,ab,kw.
49. Togo.ti,ab,kw.
50. Yemen.ti,ab,kw.
51. Zimbabwe.ti,ab,kw.
52. Cameroon.ti,ab,kw.
53. Colombia.ti,ab,kw.
54. Croatia.ti,ab,kw.

55. Guatemala.ti,ab,kw.
56. India.ti,ab,kw.
57. Indonesia.ti,ab,kw.
58. Israel.ti,ab,kw.
59. Nicaragua.ti,ab,kw.
60. Pakistan.ti,ab,kw.
61. Palestin*.ti,ab,kw.
62. Philippines.ti,ab,kw.
63. Russia.ti,ab,kw.
64. Rwanda.ti,ab,kw.
65. South Africa.ti,ab,kw.
66. Turkey.ti,ab,kw.
67. Uganda.ti,ab,kw.
68. Chechnya.ti,ab,kw.
69. or/6-68
70. 5 and 69
71. limit 70 to english language

IV. Embase

1. exp dental health/
2. exp dental procedure/
3. (oral adj3 (health or healthcare or care)).ti,ab,kw.
4. (dental adj3 (health or healthcare or care or system? or service?)).ti,ab,kw.
5. or/1-4
6. (conflict adj2 affected).ti,ab,kw.
7. post conflict.ti,ab,kw.
8. postconflict.ti,ab,kw.
9. ((fragil* or conflict* or weak or war? or war NEXT torn) adj3 (state? or countr* or territor* or region* or setting? or context? or situation?)).ti,ab,kw.
10. humanitarian*.ti,ab,kw.
11. (complex adj2 (emergency or emergencies)).ti,ab,kw.
12. Afghanistan.ti,ab,kw.
13. (Bosnia or Herzegovina).ti,ab,kw.
14. Burkina Faso.ti,ab,kw.
15. Cambodia.ti,ab,kw.
16. Central Africa?.ti,ab,kw.
17. Angola.ti,ab,kw.
18. Burundi.ti,ab,kw.
19. Cambodia.ti,ab,kw.
20. Central Africa?.ti,ab,kw.
21. Congo.ti,ab,kw.
22. Cote d'Ivoire.ti,ab,kw.
23. Ivory Coast.ti,ab,kw.
24. Djibouti.ti,ab,kw.
25. Eritrea.ti,ab,kw.
26. Ethiopia.ti,ab,kw.
27. Gambia.ti,ab,kw.

28. Guinea.ti,ab,kw.
29. Haiti.ti,ab,kw.
30. Iraq.ti,ab,kw.
31. Lao?.ti,ab,kw.
32. Lebanon.ti,ab,kw.
33. Liberia.ti,ab,kw.
34. Libya.ti,ab,kw.
35. Mali.ti,ab,kw.
36. Mozambique.ti,ab,kw.
37. Myanmar.ti,ab,kw.
38. Burma.ti,ab,kw.
39. Nepal.ti,ab,kw.
40. Niger.ti,ab,kw.
41. Nigeria.ti,ab,kw.
42. Sierra Leone.ti,ab,kw.
43. Solomon Island?.ti,ab,kw.
44. Somali*.ti,ab,kw.
45. Sudan.ti,ab,kw.
46. Syria.ti,ab,kw.
47. Tajikistan.ti,ab,kw.
48. Timor.ti,ab,kw.
49. Togo.ti,ab,kw.
50. Yemen.ti,ab,kw.
51. Zimbabwe.ti,ab,kw.
52. Cameroon.ti,ab,kw.
53. Colombia.ti,ab,kw.
54. Croatia.ti,ab,kw.
55. Guatemala.ti,ab,kw.
56. India.ti,ab,kw.
57. Indonesia.ti,ab,kw.
58. Israel.ti,ab,kw.
59. Nicaragua.ti,ab,kw.
60. Pakistan.ti,ab,kw.
61. Palestin*.ti,ab,kw.
62. Philippines.ti,ab,kw.
63. Russia.ti,ab,kw.
64. Rwanda.ti,ab,kw.
65. South Africa.ti,ab,kw.
66. Turkey.ti,ab,kw.
67. Uganda.ti,ab,kw.
68. Chechnya.ti,ab,kw.
69. or/6-68
70. 5 and 69
71. limit 70 to english language

V. Global health

1. exp Oral Health/
2. exp dental health/
3. (oral adj3 (health or healthcare or care)).mp. [mp=abstract, title, original title, heading words, cabicodes words]
4. (dental adj3 (health or healthcare or care or system? or service?)).mp. [mp=abstract, title, original title, heading words, cabicodes words]
5. or/1-4
6. (conflict adj2 affected).mp. [mp=abstract, title, original title, heading words, cabicodes words]
7. post conflict.mp. [mp=abstract, title, original title, heading words, cabicodes words]
8. postconflict.mp. [mp=abstract, title, original title, heading words, cabicodes words]
9. ((fragil* or conflict* or weak or war? or war torn) adj3 (state? or countr* or territor* or region* or setting? or context? or situation?)).mp. [mp=abstract, title, original title, heading words, cabicodes words]
10. humanitarian*.mp. [mp=abstract, title, original title, heading words, cabicodes words]
11. (complex adj2 (emergency or emergencies)).mp. [mp=abstract, title, original title, heading words, cabicodes words]
12. Afghanistan.mp. [mp=abstract, title, original title, heading words, cabicodes words]
13. Angola.mp. [mp=abstract, title, original title, heading words, cabicodes words]
14. (Bosnia or Herzegovina).mp. [mp=abstract, title, original title, heading words, cabicodes words]
15. Burkina Faso.mp. [mp=abstract, title, original title, heading words, cabicodes words]
16. Burundi.mp. [mp=abstract, title, original title, heading words, cabicodes words]
17. Cambodia.mp. [mp=abstract, title, original title, heading words, cabicodes words]
18. Central Africa?.mp. [mp=abstract, title, original title, heading words, cabicodes words]
19. Congo.mp. [mp=abstract, title, original title, heading words, cabicodes words]
20. Cote d'Ivoire.mp. [mp=abstract, title, original title, heading words, cabicodes words]
21. Ivory Coast.mp. [mp=abstract, title, original title, heading words, cabicodes words]
22. Djibouti.mp. [mp=abstract, title, original title, heading words, cabicodes words]
23. Eritrea.mp. [mp=abstract, title, original title, heading words, cabicodes words]
24. Ethiopia.mp. [mp=abstract, title, original title, heading words, cabicodes words]
25. Gambia.mp. [mp=abstract, title, original title, heading words, cabicodes words]
26. Guinea.mp. [mp=abstract, title, original title, heading words, cabicodes words]
27. Haiti.mp. [mp=abstract, title, original title, heading words, cabicodes words]
28. Iraq.mp. [mp=abstract, title, original title, heading words, cabicodes words]
29. Lao?.mp. [mp=abstract, title, original title, heading words, cabicodes words]
30. Lebanon.mp. [mp=abstract, title, original title, heading words, cabicodes words]
31. Liberia.mp. [mp=abstract, title, original title, heading words, cabicodes words]
32. Libya.mp. [mp=abstract, title, original title, heading words, cabicodes words]
33. Mali.mp. [mp=abstract, title, original title, heading words, cabicodes words]
34. Mozambique.mp. [mp=abstract, title, original title, heading words, cabicodes words]
35. Myanmar.mp. [mp=abstract, title, original title, heading words, cabicodes words]
36. Burma.mp. [mp=abstract, title, original title, heading words, cabicodes words]
37. Nepal.mp. [mp=abstract, title, original title, heading words, cabicodes words]
38. Niger.mp. [mp=abstract, title, original title, heading words, cabicodes words]
39. Nigeria.mp. [mp=abstract, title, original title, heading words, cabicodes words]
40. Sierra Leone.mp. [mp=abstract, title, original title, heading words, cabicodes words]
41. Solomon Island?.mp. [mp=abstract, title, original title, heading words, cabicodes words]
42. Somali*.mp. [mp=abstract, title, original title, heading words, cabicodes words]

43. Sudan.mp. [mp=abstract, title, original title, heading words, cabicodes words]
44. Syria.mp. [mp=abstract, title, original title, heading words, cabicodes words]
45. Tajikistan.mp. [mp=abstract, title, original title, heading words, cabicodes words]
46. Timor.mp. [mp=abstract, title, original title, heading words, cabicodes words]
47. Togo.mp. [mp=abstract, title, original title, heading words, cabicodes words]
48. Yemen.mp. [mp=abstract, title, original title, heading words, cabicodes words]
49. Zimbabwe.mp. [mp=abstract, title, original title, heading words, cabicodes words]
50. Cameroon.mp. [mp=abstract, title, original title, heading words, cabicodes words]
51. Colombia.mp. [mp=abstract, title, original title, heading words, cabicodes words]
52. Croatia.mp. [mp=abstract, title, original title, heading words, cabicodes words]
53. Guatemala.mp. [mp=abstract, title, original title, heading words, cabicodes words]
54. India.mp. [mp=abstract, title, original title, heading words, cabicodes words]
55. Indonesia.mp. [mp=abstract, title, original title, heading words, cabicodes words]
56. Israel.mp. [mp=abstract, title, original title, heading words, cabicodes words]
57. Nicaragua.mp. [mp=abstract, title, original title, heading words, cabicodes words]
58. Pakistan.mp. [mp=abstract, title, original title, heading words, cabicodes words]
59. Palestin*.mp. [mp=abstract, title, original title, heading words, cabicodes words]
60. Philippines.mp. [mp=abstract, title, original title, heading words, cabicodes words]
61. Russia.mp. [mp=abstract, title, original title, heading words, cabicodes words]
62. Rwanda.mp. [mp=abstract, title, original title, heading words, cabicodes words]
63. South Africa.mp. [mp=abstract, title, original title, heading words, cabicodes words]
64. Turkey.mp. [mp=abstract, title, original title, heading words, cabicodes words]
65. Uganda.mp. [mp=abstract, title, original title, heading words, cabicodes words]
66. Chechnya.mp. [mp=abstract, title, original title, heading words, cabicodes words]
67. or/6-66
68. 5 and 67
69. limit 68 to english language
